# SWI/SNF Alterations in Squamous Bladder Cancers

**DOI:** 10.3390/genes11111368

**Published:** 2020-11-19

**Authors:** Fabian Achenbach, Michael Rose, Nadina Ortiz-Brüchle, Lancelot Seillier, Ruth Knüchel, Veronika Weyerer, Arndt Hartmann, Ronja Morsch, Angela Maurer, Thorsten H. Ecke, Stefan Garczyk, Nadine T. Gaisa

**Affiliations:** 1 Institute of Pathology, RWTH Aachen University, Pauwelsstrasse 30, 52074 Aachen, Germany; fabian.achenbach@rwth-aachen.de (F.A.); mrose@ukaachen.de (M.R.); nortiz-bruechle@ukaachen.de (N.O.-B.); lseillier@ukaachen.de (L.S.); rknuechel-clarke@ukaachen.de (R.K.); amaurer@ukaachen.de (A.M.); sgarczyk@ukaachen.de (S.G.); 2 Institute of Pathology, University Hospital Erlangen, Friedrich-Alexander University Erlangen-Nürnberg (FAU), 91054 Erlangen, Germany; veronika.weyerer@uk-erlangen.de (V.W.); Arndt.Hartmann@uk-erlangen.de (A.H.); 3 Department of Urology, RWTH Aachen University, 52074 Aachen, Germany; romorsch@ukaachen.de; 4 Department of Urology, Helios Clinic, 15526 Bad Saarow, Germany; thorsten.ecke@helios-gesundheit.de

**Keywords:** SWI/SNF complex, ARID1A, squamous bladder cancer, immune checkpoint inhibitors

## Abstract

Dysfunction of the SWI/SNF complex has been observed in various cancers including urothelial carcinomas. However, the clinical impact of the SWI/SNF complex in squamous-differentiated bladder cancers (sq-BLCA) remains unclear. Therefore, we aimed to analyze potential expression loss and genetic alterations of (putative) key components of the SWI/SNF complex considering the co-occurrence of genetic driver mutations and PD-L1 expression as indicators for therapeutic implications. Assessment of *ARID1A*, *SMARCA2*, *SMARCA4*, *SMARCB1/INI1*, *SMARCC1*, *SMARCC2* and *PBRM1* mutations in a TCGA data set of sq-BLCA (*n* = 45) revealed that *ARID1A* was the most frequently altered SWI/SNF gene (15%) while being associated with protein downregulation. Genetic alterations and loss of ARID1A were confirmed by Targeted Next Generation Sequencing (NGS) (3/6) and immunohistochemistry (6/116). Correlation with further mutational data and PD-L1 expression revealed co-occurrence of ARID1A loss and *TP53* mutations, while positive correlations with other driver mutations such as *PIK3CA* were not observed. Finally, a rare number of sq-BLCA samples were characterized by both ARID1A protein loss and strong PD-L1 expression suggesting a putative benefit upon immune checkpoint inhibitor therapy. Hence, for the first time, our data revealed expression loss of SWI/SNF subunits in sq-BLCA, highlighting ARID1A as a putative target of a small subgroup of patients eligible for novel therapeutic strategies.

## 1. Introduction

In 2018, bladder cancer was the 10th most common cancer worldwide, with estimated 549,000 new cases and 200,000 deaths. It is more common in men than in women, with respective incidence and mortality rates of 9.6 and 3.2 per 100,000 men, about four times those of women globally [1]. Furthermore, bladder cancer is the second most common genitourinary malignancy [2]. The most significant risk factor is age and the median age at diagnosis is 70 years [3]. Because of the demographic change, it can be assumed that the number of new cases will increase in the near future. Over 90% of bladder cancers are urothelial carcinomas with distinct molecular characteristics for muscle-invasive bladder cancers (MIBCs) such as *TP53* mutations or non-muscle-invasive bladder cancers (NMIBCs) including activating *FGFR3* mutations or *PIK3CA* alterations [3]. Only 5% of all bladder cancers are squamous cell carcinomas (SCCs) [4] characterized by low frequent alterations of *ERBB* genes but frequent *TP53* mutations, while alterations of the *FGFR3* gene are rare but associated with worse patients’ outcome [5,6]. SCCs are mostly diagnosed in the seventh decade of life and are commonly associated with poor prognosis. Usually SCC presents at an advanced stage with an average death within 1 to 3 years [7,8]. The leading cause of SCC worldwide is a chronic infection with Schistosoma haematobium. While SCC is the predominant histological type of bladder cancer in countries where schistosomiasis is endemic—such as Egypt, Algeria, Uganda or Zimbabwe—it is rare in western countries. However, due to preventive measures and improved treatment of bilharziosis, the number of SCC in endemic areas also has decreased within the past 20 years [9]. In western countries, chronic infections and irritations appear to be the main cause of non-schistosomiasis-associated SCC. It occurs in patients with chronic inflammatory disorders of the bladder, persistent calculi, chronic cystitis and bladder diverticuli [10].

Switch/sucrose-nonfermenting (SWI/SNF) complexes are members of the chromatin-remodeling family. To date, 29 components have been identified involved in assembling three different SWI/SNF complexes—i.e., the canonical BRG1/BRM-associated factor (cBAF), the polybromo-associated BAF (PBAF) and the recently described non-canonical BAF (ncBAF) complex [11,12]. While distinct subunits are commonly found in all three SWI/SNF protein complexes (referred to as “shared”—e.g., SMARCA4) or at least part of the BAF and/or PBAF complex (e.g., SMARCC2), there are also subcomplex-specific members such as ARID1A (BAF-specific) [11,13]. In general, SWI/SNF complexes play a central role in cellular processes such as transcription, cell cycle control, proliferation, differentiation and repair of DNA lesions [14]. Furthermore, mutations and loss of expression of central SWI/SNF proteins were found in over 20% of different neoplasms, such as oesophageal adenocarcinoma, lung cancer, ovarian clear cell and endometrioid cancers as well as uterine endometrioid carcinomas [14,15,16,17]. Underlying mechanisms are not fully understood; however, several components of the SWI/SNF are thought to function as tumor suppressors [18]. In addition, loss of SWI/SNF subunits, in particular of ARID1A, is of interest with regard to the development of novel therapeutic strategies [19,20,21,22,23,24,25,26,27] mostly based on synthetic lethality—i.e., affecting only those cancer cells that are characterized by functional loss of two genes leading to cell death, whereas individual alterations alone are compatible with viability [28,29].

Among the identified subunits to date, the AT-rich interactive domain-containing protein 1A (ARID1A) is the most frequently mutated SWI/SNF component in urothelial bladder cancer [30]. Loss of ARID1A expression is associated with higher stage and more aggressive variants of urothelial carcinomas. Therefore, low expression of ARID1A appears to be an indicator of poor survival [31]. The role of SWI/SNF in squamous cell carcinomas of the urinary bladder has not yet been investigated. Since standard chemo- or immunotherapy for advanced, metastasized squamous bladder cancer is of limited success, the knowledge of SWI/SNF alteration-mediated therapeutic vulnerabilities might offer a chance to develop more effective therapeutic strategies. 

## 2. Materials and Methods

### 2.1. Patient Samples and Tissue Microarrays 

Squamous differentiated bladder cancers were retrospectively collected from pathology archives of the German Study Group of bladder cancer (*n* = 68 pure, *n* = 48 Mixed) over 17 years (1998–2015). For cohort characteristics see Table 1. Tissue microarrays of formalin-fixed paraffin-embedded (FFPE) surgical specimens were used as previously described [32,33,34]. The RWTH University Hospital Aachen local ethics committee approved the retrospective, pseudonymized study of archival tissues (RWTH EK 009/12). 

### 2.2. Immunohistochemistry

Overall, TMAs used in this study comprise two 1.5 mm cores of different tumor areas for each patient tumor sample. For anti-ARID1A stainings, TMA sections were pretreated with DAKO PT-Link heat induced antigen retrieval with high pH (pH9) Target Retrieval Solution (DAKO, Hamburg, Germany), incubated for 60 min at room temperature with anti-ARID1A (1:250, D2A8U, Cell Signaling) as previously specified [30]. An EnVision FLEX/HRP detection system and counterstaining with EnVision FLEX Hematoxylin were applied. For stainings of further components of the SWI/SNF complex, the BenchMark ULTRA system (Ventana Medical Systems Inc, 1910 Innovation Park Drive, Tucson, AZ, USA) and antibodies against the following antigens were used: anti-SMARCB1 (INI1) (ZSI1, 1:50, Zytomed), anti-SMARCA2 (polyclonal antibody, 1:100, Atlas Antibodies AB, Stockholm, Sweden), anti-SMARCA4 (anti-BRG1 antibody, clone EPNCIR111A, 1:100, Abcam; Cambridge, UK), anti-SMARCC1 (HPA026853, 1:50, Atlas Antibodies AB), anti-SMARCC2 (HPA021213, 1:50, Atlas Antibodies AB) and anti-PBRM1 (clone CL0331, dilution 1:50, Atlas Antibodies AB). Immunohistochemical stainings were evaluated by two medical doctors/one senior specialist in uropathology (F.A. and N.T.G.). Staining intensities (0 = no staining, 1 = weak staining, 2 = moderate staining, 3 = strong staining) and percentages of positive stained viable tumor cells according to the system of Remmele and Stegner were reported [35]. Strong nuclear staining of accompanying stromal fibroblasts, inflammatory cells, vascular endothelial cells or normal epithelial cells served as internal positive control. If normal (inflammatory) cells showed only weak expression, immunohistochemical staining was repeated on a second TMA section. Finally, cases with absent or very weak staining in normal lymphocytes after repeated staining were excluded.

### 2.3. DNA Extraction 

DNA extraction of FFPE tissue samples (*n* = 69 samples) was performed using a QIAamp DNA Mini Kit (Qiagen, Hilden, Germany) as previously described [36]. For Targeted Next Generation Sequencing (NGS) analyses, DNA was isolated from FFPE tissue (*n* = 6) using the automated Maxwell 16 system and corresponding FFPE Tissue LEV DNA Purification Kit (Promega, Mannheim, Germany) according to the manufacturer’s instructions. 

### 2.4. SNaPshot and Sanger Sequencing

*PIK3CA* and *FGFR3* mutational analyses were performed using the SNaPshot method for the simultaneous detection of hotspot mutations according to Hurst et al. [37] and as described previously [38]. PCR-amplification and Sanger sequencing was performed for *TP53* and *CDKN2A* as specified in 2016 [6]. For details of primer sequences and PCR conditions see Supplementary Table S1. 

### 2.5. Targeted Next Generation Sequencing (NGS) Analysis

Targeted NGS was conducted for *n* = 6 samples to evaluate the ARID1A, SMARCA4 and SMARCB1 mutation status. Tumor cellularity was >20% for all samples. For library preparation, between 45 and 55 ng DNA were used. However, DNA quality assessment (GeneRead DNA QuantiMIZE Assay Kit, Qiagen, Hilden, Germany) revealed sufficient quality for only five samples. Libraries were generated using the AmpliSeq for Illumina Comprehensive Panel v3 (Illumina, San Diego, USA; reference genome hg19), according to the manufacturer’s instructions. Normalized libraries were sequenced on a NextSeq 500 platform (Illumina, San Diego, CA, USA) using a NextSeq 500/550 Mid Output kit (2 × 150 cycles; Illumina). Bam-file generation was performed with the DNA amplicon module version 1.0.2.1415 (Illumina, San Diego, CA, USA). Single-nucleotide variant analysis was conducted using Sequence Pilot Software version 5.1.0 (SeqNext module; JSI Medical Systems, Ettenheim, Germany). A virtual panel was created to analyze the coding and adjacent intronic regions of ARID1A, SMARCA4 and SMARCB1 (reference sequences: ARID1A: HGNC:11110, RefSeq: NM_006015.6; SMARCA4: HGNC:11100, RefSeq: NM_003072.3; SMARCB1: HGNC:11103, RefSeq: NM_003073.4). Changes with an allele frequency above 10% were taken into account if not already classified as known artifacts for the panel. Further variant filtering was conducted as follows: Missense variants with an allele frequency >2% in the normal population (according to 1000 Genomes (http://www.internationalgenome.org, last accessed 2nd September 2019) or dbSNP v153 (https://www.ncbi.nlm.nih.gov/snp, last accessed 27 September 2019)) and non-splicing–relevant silent, untranslated region (UTR) and intronic variants not affecting the canonical splice-site were considered benign. Additionally, we excluded missense variants classified as benign or likely benign in the ClinVar database (https://www.ncbi.nlm.nih.gov/clinvar, last accessed 27 September 2019). A possible effect of mutations on splicing was determined using Alamut software (Alamut VISUAL v2.14.0, SOPHiA interactive biosoftware, Rouen, France) (included splicing predictions: SpliceSiteFinder-like, MaxEntScan, NNSPLICE 0.9, GeneSplicer; for detailed information see software documentation). For *ARID1A*, *SMARCA4* and *SMARCB1* copy number variation (CNV) analysis, an in-house algorithm (validated using three NGS panels; >150 samples) was used. The ACopy tool is based on an exponential growth model for amplification of PCR products [39].

### 2.6. Analysis of the TCGA Sq-BLCA Data Set

To identify mutations for seven genes of the SWI/SNF-complex of SCC in the TCGA-BLCA cohort [40] we extracted the patient IDs for samples classified as “NOS with squamous differentiation” (*n* = 42) and “Squamous cell carcinoma” (*n* = 3) based on the pathologic classification described by Robertson et al. [40]. No further publicly available platforms providing additional squamous bladder cancer data sets exist. Not without reason, in particular pure, non-schistosomiasis-associated SCC is a rare disease in the western world, with very low incidence rates (e.g., 0.6–1.2 per 100,000 person-years) [41]. Assessment of genetic alterations and the mutation spectrum was performed using cBioPortal (https://www.cbioportal.org/, [42,43]) filtering for the extracted patient IDs. 

### 2.7. Statistical Analysis 

Statistical analysis was performed using Statistical Package for the Social Sciences (SPSS) software version 26.0 (SPSS inc., Chicago, IL, USA). *p*-values < 0.05 were considered significant. Statistical associations between clinico-pathological and molecular factors were determined by Fisher’s exact test. Survival curves for recurrence-free (RFS) and overall survival (OS) were calculated using the Kaplan–Meier method with log-rank statistics. RFS/OS was measured from surgery until relapse/death and was censored for patients alive without evidence of relapse/death at the last follow-up date. Correlation analysis was performed by calculating a Spearman’s rank correlation coefficient. The Expression correlation network was plotted using the R package “Rgraphviz” [44], and significant correlations (Spearman correlation coefficient, *p* < 0.05) between subunits were plotted as edges. 

## 3. Results

### 3.1. Analysis of Frequently Altered Subunits of the BAF and PBAF SWI/SNF Complexes in TCGA Sq-BLCA 

**Figure 1:**
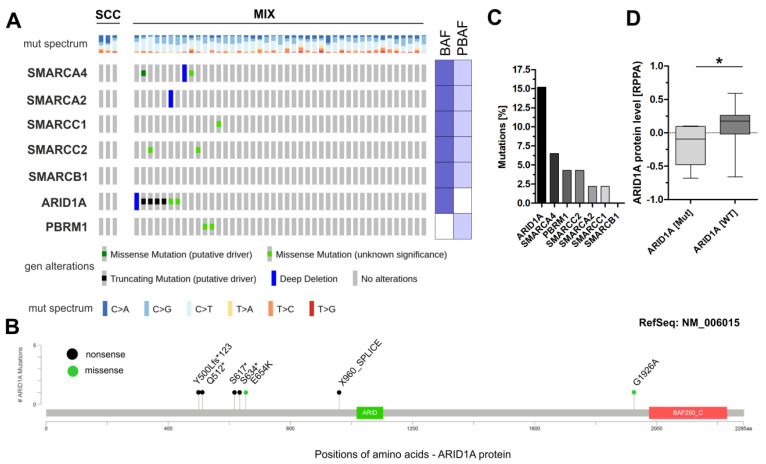
SWI/SNF alterations and ARID1A protein expression in the sq-BLCA cohort of the TCGA network. (**A**) Genetic alterations of different subunits of the SWI/SNF complex (including missense mutations, truncating mutations, amplifications and deep deletions). (**B**) Mutation Mapper illustrating positions of identified *ARID1A* alterations (nonsense and missense) relative to the protein sequence and domains (green and red box). Protein domains are indicated according to PFAM: green box: ARID domain; red box: BAF250_C domain. (**C**) Mutational frequencies of analyzed subunits of the SWI/SNF complex. (**D**) Box plots illustrating ARID1A protein expression classified by *ARID1A* mutations. BAF (dark blue): BRG1/BRM-associated factor; PBAF (light blue): polybromo-associated BAF; MUT: mutated; WT: wildtype. * *p* < 0.05.

In order to give first insights into the mutational status of (putative) key components of the SWI/SNF complex in squamous bladder cancers (for study design see Supplementary Figure S1), carcinomas with histologically squamous differentiation (*n* = 3 pure SCC and *n* = 42 MIX) of The Cancer Genome Atlas (TCGA) were analyzed for genetic alterations of seven frequently affected subunits of the SWI/SNF complexes BAF and PBAF [11,12]. Alterations of the BAF-specific component *ARID1A* were the most frequent events (15.2%, 7/46), comprising one deep deletion, four truncating mutations and two missense mutations indicating impaired protein function (Figure 1A,B). 

Only low mutation frequencies were observed in sq-BLCA for components potentially involved in assembly of both complexes—i.e., BAF and PBAF: *SMARCA4* 6.5% (3/46), *SMARCC2* 4.3% (2/46), *SMARCA2* 2.2% (1/46), *SMARCC1* 2.2% (1/46) and *SMARCB1* 0% (0/46). The gene encoding the PBAF-specific subunit *PBRM1* was mutated in 4.3% of samples (2/46) (Figure 1A). Determining the ARID1A protein level in dependency of its mutational status, we confirmed a significantly lower expression in tumors with genetic alterations of the *ARID1A* gene including missense mutations (Figure 1C)—i.e., frequent *ARID1A* mutations correlate with loss of ARID1A protein in TCGA Sq-BLCA. 

As genetic alterations of SWI/SNF components are known to be associated with patients’ outcome, we correlated SWI/SNF mutations with clinico-pathological parameters and analyzed recurrence-free (RFS) and overall survival (OS) as an indicator of potential prognostic impact. We focused on patients with at least one genetic alteration in one or more of the analyzed components as well as on those harboring *ARID1A* mutations (missense vs. nonsense). Using a Fisher’s exact test, no associations of SWI/SNF mutations or *ARID1A* mutations with clinico-pathological characteristics were observed (Supplementary Tables S2–S4). Kaplan–Meier analysis did not show any association of mutated SWI/SNF components and/or *ARID1A* mutations with RFS and OS (Supplementary Figure S2). 

### 3.2. Immunohistochemical Analysis of Frequently Altered Subunits of the SWI/SNF-Complex in an Independent Squamous Bladder Cancer Cohort 

**Figure 2:**
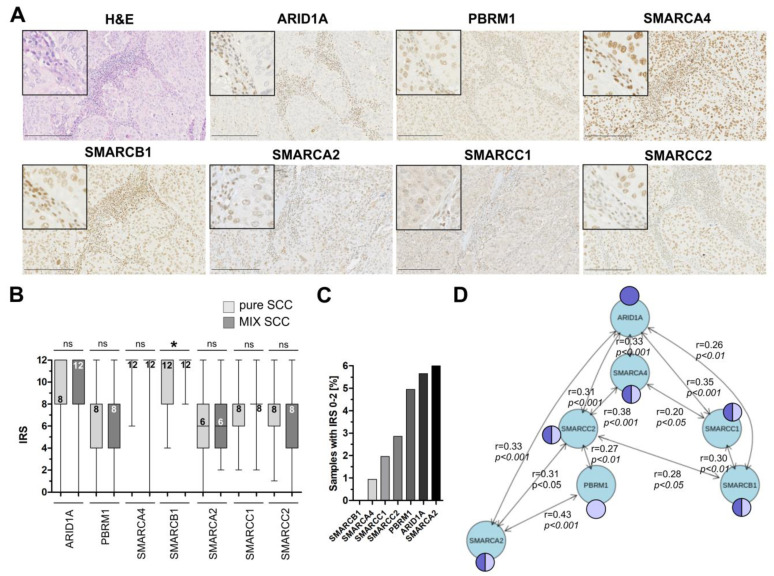
Protein expression of subunits of the SWI/SNF complexes BAF and PBAF in squamous-differentiated bladder cancers (sq-BLCA). (**A**) H&E and immunohistochemical staining of seven subunits of the SWI/SNF complex are shown for a representative tissue core with *ARID1A* mutation (c.4005-2A>G, p.(?)). Black scale bar: 100 µM. For further immunohistochemical ARID1A staining according to the range of Immune Reactive Scores (IRSs) see Supplementary Figure S3. (**B**) Box plot graphs show overall distribution of IRS staining results of subunits for urothelial cancers with squamous components (MIX SCC) and pure squamous cancers (SCC). The numeric values correspond to the median value. (**C**) Frequencies of expression loss (IRS ≤ 2) shown for all analyzed subunits. (**D**) Expression network illustrating the statistical correlation between analyzed subunits of BAF and PBAF. Only significant (*p* ≤ 0.05) spearman correlations (r-values as indicated) are shown. Dark blue: component of the BAF complex; light blue: component of the PBAF complex; * *p* < 0.05.

Next, *n* = 116 samples of patients with pure squamous cell carcinoma (*n* = 68) and mixed urothelial carcinoma with substantial squamous differentiation (*n* = 48) were analyzed for seven SWI/SNF complex proteins (ARID1A, SMARCA4, SMARCB1, SMARCC1, SMARCC2, SMARCA2 and PBRM1) by immunohistochemistry (Figure 2A). In total, 68.1% of the carcinomas presented as high-grade cancers, while except for one sample, all bladder tumors showed muscle-invasion (for cohort characteristics see Table 1). 

Based on an adapted Immune Reactive Score (IRS) by Remmele and Stegner [35], a semiquantitative score (0–2 = negative; 3–12 = positive) was applied. Considering the different subunits of the SWI/SNF-complex which have been previously shown to be frequently altered in various cancer entities including urothelial carcinomas [13], we found no significant differences between pure and mixed squamous carcinomas regarding the median expression for ARID1A (median IRS SCC: 8 (*n* = 64); median IRS MIX: 12 (*n* = 45)), SMARCA4 (median IRS SCC: 12 (*n* = 62); median IRS MIX: 12 (*n* = 44)), SMARCC1 (IRS SCC: 8 (*n* = 61); IRS MIX: 8 (*n* = 41)), SMARCC2 (IRS SCC: 8 (*n* = 62); IRS MIX: 8 (*n* = 43)), SMARCA2 (IRS SCC: 6 (*n* = 57); IRS MIX: 6 (*n* = 43)) and PBRM1 (IRS SCC: 8 (*n* = 56); IRS MIX: 8 (*n* = 45)) (Figure 2B). Expression of SMARCB1 significantly differs (*p* < 0.05) between pure SCC and MIX-SCC ranging between IRS 4–12 for SCC (*n* = 62) and 8–12 for MIX tumors (*n* = 42). However, the median expression did not differ (IRS SCC: 12; IRS MIX: 12) (Figure 2B). Focusing on those candidates lacking expression (IRS 0–2), 6% of our squamous bladder cancers were identified to show loss of expression of SMARCA2, followed by ARID1A (5.5%), PBRM1 (5.0%), SMARCC2 (2.9%), SMARCC1 (2.0%) and SMARCA4 (0.9%) (Figure 2C). For detailed data of expression loss see Table 2.

As previous studies revealed involvement of distinct components in assembly of different SWI/SNF complexes [11,12], we further analyzed statistical associations between expression of subunits known to be potentially present in both protein complexes (either BAF and/or PBAF) as well as the BAF- (ARID1A) and PBAF-specific (PBRM1) components using a non-parametric Spearman-rank correlation. A frequent and significant similarity of ARID1A expression with five BAF-associated subunits was observed in our cohort (Figure 2D).

PBRM1 (PBAF-specific complex) correlated with two PBAF-associated subunits. Contrary to that, ARID1A loss correlated with lack of expression of the BAF subunits SMARCA4 (Spearman r: 0.438, *p* ≤ 0.001), SMARCC1 (Spearman r: 0.492, *p* ≤ 0.001) and SMARCC2 (Spearman r: 0.296, *p* ≤ 0.01). No correlation was found between ARID1A and PBRM1, SMARCA2 or SMARCB1.

### 3.3. Correlation of ARID1A Expression Loss with Clinico-Pathological Parameters and Known Genetic Drivers

**Figure 3:**
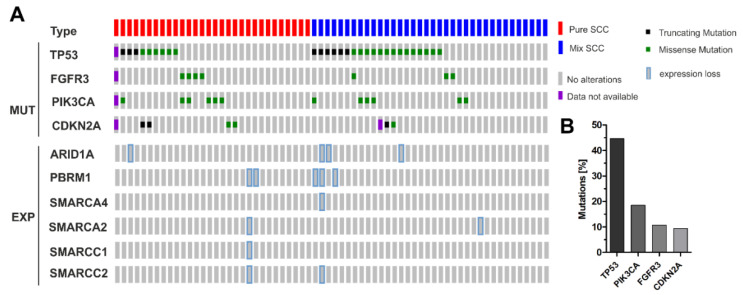
Expression loss of SWI/SNF subunits in sq-BLCA with known genetic driver mutations. (**A**) Upper lines: mutational spectrum of genes (*TP53, FGFR3, PIK3CA, CDKN2A*) potentially involved in bladder cancer development and progression (SCC: *n* = 30; MIX SCC: *n* = 36). *TP53* was the most frequently mutated gene in pure SCC (9/29) as well as MIX-SCC (20/36), while *FGFR3* (SCC: 4/29; MIX: 3/36) and *CDKN2A* (SCC: 4/29; MIX: 2/35) mutations were less abundant (SCC: 4/29; MIX: 3/36). *PIK3CA* driver mutations were observed in 6/29 (SCC) and in 6/36 (MIX) tumors. Lower lines: corresponding expression loss of subunits of the SWI/SNF complex. MUT: mutations; EXP: expression. (**B**) Overall mutational frequencies of analyzed driver genes.

Next, we tested associations between expression loss of analyzed subunits with both clinico-pathological characteristics and driver mutations as indicators for prognostic risk and therapeutic implication (Figure 3A,B). Novel *PIK3CA* mutational analyses were performed by SNaPshot according to Hurst et al. [37], while PCR-amplification and Sanger sequencing was performed for *CDKN2A* as specified in 2016 [6]. All findings were correlated including previously published mutational data for *TP53* and *FGFR3* [5]. With the exception of ARID1A, neither associations between expression loss and clinico-pathological parameters—such as age at diagnosis, tumor size and histological tumor type - nor driver mutations were found. ARID1A expression did not significantly correlate with clinico-pathological parameters (Table 3). Please note that the sample number showing ARID1A expression loss is limited, which could affect statistical accuracy. However, all six tumors that did not express ARID1A were diagnosed in advanced stages—i.e., *n* = 5 pT3, *n* = 1 pT4, *n* = 2 with positive lymph node status, and *n* = 4 with high-grade differentiation. ARID1A expression was further significantly associated with *TP53* mutations (*p* < 0.05) (Table 3). Interestingly, ARID1A expression loss was not observed in tumors with genetic alterations of *FGFR3* or *PIK3CA*.

### 3.4. ARID1A Protein Loss Overlaps with Genetic ARID1A Alterations and PD-L1 Expression in the Independent Squamous Bladder Cancer Cohort 

**Figure 4:**
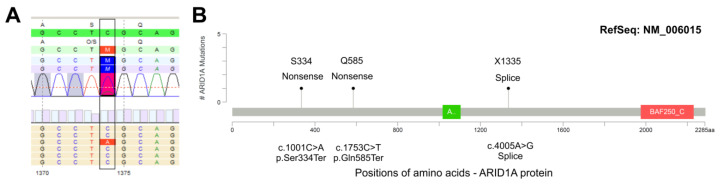
*ARID1A* mutations in sq-BLCA. (**A**) Exemplary illustration of a nonsense *ARID1A* mutation with an allele frequency of 20% (c.1001C>T, p.Ser334Ter, estimated tumor cell content 40%). (**B**) Summarized *ARID1A* mutations identified in Sq-BLCA with clear expression loss (IRS < 2). Protein domains are indicated according to PFAM: green box: ARID domain; red box: BAF250_C domain.

Since ARID1A loss seems to be associated with advanced tumor stages, we focused on this important SWI/SNF component with therapeutic potential to confirm that ARID1A protein loss results from genetic *ARID1A* gene alterations. Six tumors (*n* = 4 MIX, *n* = 2 SCC) with loss of ARID1A expression (Figure 4) were sequenced by NGS. Three out of the six ARID1A-deficient samples showed *ARID1A* mutations (c.1001C>A, p.(Ser334Ter), allele frequency (AF) 20%; c.1753C>T, p.(Gln585Ter), AF 23%; c.4005-2A>G, p.(?), canonical splice site, AF 27%). No high-level copy number alteration was detected for *ARID1A*.

To our knowledge, none of the three mutations has been previously described in the literature, but all three mutations are annotated in the COSMIC database v92 (https://cancer.sanger.ac.uk/cosmic; c.1001C>T (p.Ser334Ter/COSM6983737): *n* = 1, pancreatic carcinoid-endocrine tumor [P-0012246-T01-IM5]; c.1753C>T (p.Gln585Ter/COSM1133047): *n* = 2, breast carcinoma [H_KU-1186-1186_A2_core], Burkitt lymphoma BL-6; c.4005-2A>G (COSM6925751): *n* = 1, transitional cell bladder carcinoma [P-0003024-T01-IM3]). Two of the three mutations are also (redundantly) listed in the cBioPortal database v3.4.13 (https://www.cbioportal.org/; c.1001C>T: *n* = 1, pancreatic neuroendocrine tumor [P-0012246-T01-IM5], classification: likely oncogenic; c.4005-2A>G: *n* = 1, bladder urothelial carcinoma [P-0003024-T01-IM3], classification: likely oncogenic). The mutation c.4005-2A>G affects the canonical splice site and leads to loss of the acceptor splice site according to distinct prediction tools. All three mutations are, therefore, most likely deleterious and probably lead to a nonsense-mediated decay of the truncated protein and, therefore, to a protein loss. 

It is thought that *ARID1A*-mutated cancers may cooperate with immune checkpoint blockade therapy [19], thus providing novel therapeutic strategies for cancer management. As we recently showed that PD-L1 was frequently expressed in squamous bladder cancer [45], ARID1A alterations were correlated with expression of PD-L1 using the 28-8 antibody clone as an indicator of immune checkpoint inhibitor (ICI) treatment access. According to current European Medicines Agency (EMA)-approved guidelines for first line therapy of bladder cancer with pembrolizumab (CPS ≥ 10) and atezolizumab (IC-score ≥ 2/IC ≥ 5%), a single *ARID1A*-mutated cancer (1/3) was identified to be potentially eligible for atezolizumab first line therapy (Supplementary Table S5). Considering protein loss of ARID1A, another case was revealed—i.e., overall two SCC/MIX specimens of the urinary bladder were characterized by ARID1A protein loss and strong PD-L1 expression (IC-score ≥ 2/IC ≥ 5%) suggesting a putative synergistic impact and improved ICI therapy success similar to recent studies in urothelial cancers [24]. 

## 4. Discussion

To date, dysfunction of the components of the SWI/SNF complex has been shown for various cancer entities [17] including urothelial cancer [13]. TCGA data demonstrate that *ARID1A* is among the most frequently mutated genes among different types of cancer, such as stomach adenocarcinomas (18–31%) or uterine corpus endometrioid carcinomas (34%) [46]. Most *ARID1A* mutations are inactivating truncating mutations [46]—e.g., 63% of *ARID1A* gene alterations in urothelial carcinomas [30] or over 90% of *ARID1A* mutations in ovarian clear cell carcinoma [47]. *ARID1A* mutated carcinomas are associated with poor prognosis, and for instance, in breast cancer patients, inactivated *ARID1A* suggests a tumor suppressive function [48,49]. 

Recently, we revealed frequent genetic alterations of genes encoding for SWI/SNF subunits including *ARID1A* with a frequency of 26% in urothelial bladder cancer [30]. Most of these mutations, in particular truncating alterations, are likely associated with a functional loss of proteins. In line with this, we identified *ARID1A* mutations in 15% of sq-BLCA of the TCGA data set. The pathological/functional significance of identified missense mutations remains elusive; however, by stratifying the mutations we significantly observed reduced ARID1A protein levels for both—i.e., for nonsense mutations as well as for the combined group of nonsense and missense mutations. Further mechanisms potentially involved in gene silencing such as epigenetic silencing or mutations in non-coding regions as well as post-transcriptional or translational modifications [50] might be likely. Wu and colleagues showed, for instance, that heterozygous *ARID1A* mutations correlated with loss of protein expression—i.e., 73% of tumors with heterozygous *ARID1A* mutations lacked protein expression [48]—suggesting a second hit on the remaining allele. Considering that, we confirmed missense and nonsense mutations of *ARID1A* in both pure and mixed SCC samples which were characterized by ARID1A protein loss. Interestingly, we found that protein loss of the BAF-specific subunit ARID1A was closely associated with expression loss of the commonly shared and central subunits SMARCA4 and SMARCC1, as well as with the BAF/PBAF-associated factor SMARCC2. In turn, ARID1A expression correlates with all analyzed components potentially involved in assembly of the BAF complex [11,12] suggesting a predominant role of this canonical SWI/SNF complex in SCC. However, we are aware that a statistical correlation does not provide the exact protein interaction in single cells. In addition, residual subunits could be of importance to compensate missing factors, thus maintaining the SWI/SNF activity as, for instance, already demonstrated for the catalytic subunits SMARCA4 and SMARCA2. Both subunits have been shown to be mutually exclusive subunits in SWI/SNF complexes, and survival of *SMARCA2*-mutated cells depends on the residual SMARCA4-containing complex activity in specific tumor entities [51]. Thus, future studies addressing the role and function of the different SWI/SNF complexes and their corresponding subunits are required to decipher the mechanisms behind this in sq-BLCA. 

Besides involvement of ARID1A in SW/SNF-mediated chromatin remodeling, ARID1A is thought to contribute to DNA damage repair, especially DNA double strand break (DSB) repair [50]. It has been shown that suppression of ARID1A led to a higher cellular sensitivity to cisplatin due to higher rates of DSB, triggered by deficient DNA repair [52]. In endometrial carcinomas, *ARID1A* mutations are associated with mismatch repair deficiency and normal p53 expression [53]. Bosse and colleagues showed a nearly mutual exclusivity of ARID1A loss and mutant-like TP53 expression, while alterations of the PI3K-AKT pathway were more frequent when ARID1A expression was lost [54]. Coexistence of *PIK3CA* and *ARID1A* mutations has been shown before [55], whereas association of both events was not observed in our SCC/MIX samples of the urinary bladder. We are aware of the potential bias due to the low number of *PIK3CA* mutations; however, none of the tumors lacking ARID1A expression showed evidence for any of the analyzed driver mutations (i.e., *PIK3CA* and *CDKN2A*). Thus, a hypothesized causal and functional link between both events (e.g., *PIK3CA* and ARID1A mutations/expression) seems unlikely in squamous bladder cancer. In turn, ARID1A loss occurred in a *TP53*-deficient genetic background suggesting a regulation of potentially different biological processes to those described to date [56], but affecting cell cycle control which should be further studied in more detail in the future. 

However, accumulating studies propose the involvement of functional ARID1A loss in synthetic lethality, which contributes to the response to various classical [20,21] and novel therapeutic options, including immune checkpoint inhibitors (ICI) [19,22,23]. Goswami and colleagues have recently shown that *ARID1A* mutation in combination with immune cytokine CXCL13 expression predicts response to immune checkpoint inhibitors in metastasized bladder cancers [24]. As we already provided a rationale for ICI treatment of SCC of the urinary bladder [45], ARID1A protein loss may predict increased efficiency of ICI therapy. However, a correlation between *ARID1A* mutations and increased PD-L1 expression as previously reported [19] could not be confirmed in sq-BLCA. The co-occurrence was rare, and only a subgroup of patients with *ARID1A* mutations may benefit from ICI treatment. In addition, Fukumoto and colleagues showed that inhibition of histone deacetylase 6 (HDAC6) contributes to growth suppression of *ARID1A*-mutated tumors, while synergistic effects were shown in combination with anti-PDL1 therapy [25]. Although the clinical results of HDAC-inhibitors have generally been disappointing in the past [26], current studies indicate a specific targeted benefit applying HDAC-inhibitors in tumors with ARID1A loss [25,27]. Thus, ARID1A might be used as an additional biomarker for clinical response to both HDAC inhibition and anti-PD-L1 therapy, albeit its function as a biomarker has only been described for patients with advanced urothelial carcinoma yet [27]. Further clinical trials may be necessary to prove the possible synergistic effect of both HDAC- and PD-L1-inhibitors on squamous bladder cancer cells with *ARID1A* mutation.

In conclusion, we provide, for the first time, data describing expression loss of components of the SWI/SNF-complex in sq-BLCA including pure SCC, highlighting ARID1A as an interesting target for a small subgroup of patients which may benefit from novel therapeutics in an ARID1A mutated background.

## Figures and Tables

**Table 1:** Clinicopathological and demographical characteristics of the study cohort.

	Categorization	∑	n SCC	n MIX-SCC
Parameter:				
**Age at diagnosis**	median 67.5 years			
	(range 33–91 years)			
	≤67.5 years	59	38	21
	>67.5 years	57	30	27
**Gender**	Female	59	34	23
	Male	56	33	25
	unknown	1	1	0
**Histological tumor grade**	G1	1	1	0
	G2	34	25	9
	G3	77	40	37
	G4	2	0	2
	unknown	2	2	0
**Tumor stage**	pTx	5	5	0
	pT1	1	1	0
	pT2	16	12	4
	pT3	76	37	39
	pT4	18	13	5
**Lymph node status**	Negative (pN0)	72	41	31
	Positive (pN1 + pN2)	22	11	11
	unknown	22	16	6

**Table 2:** Loss of SWI/SNF proteins in squamous bladder cancer.

	SCC		MIX	
	neg	pos	neg	pos
ARID1A	2	62	4	41
PBRM1	2	54	3	42
SMARCC1	1	60	1	40
SMARCC2	2	60	1	42
SMARCA2	4	53	2	41
SMARCA4	0	62	1	43
SMARCB1	0	62	0	42

**Table 3:** Clinico-pathological parameters and known driver mutations in relation to ARID1A expression loss.

		ARID1A Expression ^b^			
		*n* ^a^	0–2	3–12	*p*-Value ^c^
***Parameter:***					
Age at diagnosis					
	median age: 67 years				
	≤67 years	56	3	53	0.954
	>67 years	53	3	50	
Gender					
	female	55	3	52	0.963
	male	53	3	50	
Histological tumor grade ^d^					
	G1-G2	30	2	28	0.816
	G3	73	4	69	
Tumor stage ^d^					
	pT1-pT2	13	0	13	0.328
	pT3-pT4	86	6	80	
Lymph node status					
	neg	65	4	61	0.600
	neg	21	2	19	
*TP53* mut ^e^					
	neg	30	0	30	0.030
	neg	27	4	23	
*FGFR3* mut ^e^					
	neg	50	4	46	0.442
	neg	7	0	7	
*CDKNA2* mut ^e^					
	neg	51	4	47	0.520
	neg	5	0	5	
*PIK3CA* mut ^e^					
	neg	45	4	41	0.288
	pos	12	0	12	

^a^ Only patients with primary bladder cancer were included: ^b^ IRS according to Remmele [35]; ^c^ Fisher’s exact test; ^d^ according to WHO 2004 classification; ^e^ due to limited availability of material, experimental data, case numbers vary for different methods as indicated. Significant *p*-values are marked in bold face.

## Supplementary Materials

The following are available online at https://www.mdpi.com/2073-4425/11/11/1368/s1. The datasets supporting the conclusions of this article are included within the article and its additional files: Supplementary Table S1: Primer sequences for Sanger sequencing of FFPE Material. Supplementary Tables S2–S4: Clinico-pathological parameters of the TCGA sq-BLCA data set in relation to SWI/SNF/ARID1A mutations. Supplementary Table S5: Overlap of ARID1A mutations/expression loss with PD-L1 expression. Supplementary Table S6: Abbreviation list. Supplementary Figure S1: Study design of the project. Supplementary Figure S2: Prognostic impact of SWI/SNF and ARID1A mutations on tumor patients’ survival. Supplementary Figure S3: Immunohistochemical staining of ARID1A according to calculated IRS.
