# Supplementary material for: SWI/SNF Alterations in Squamous Bladder Cancers
Source: Genes (Basel). 2020 Nov 19;11(11):1368. doi: 10.3390/genes11111368 (PMC7699259; doi:10.3390/genes11111368)
Supplement: Supplementary file 1 [file genes-11-01368-s001.pdf]

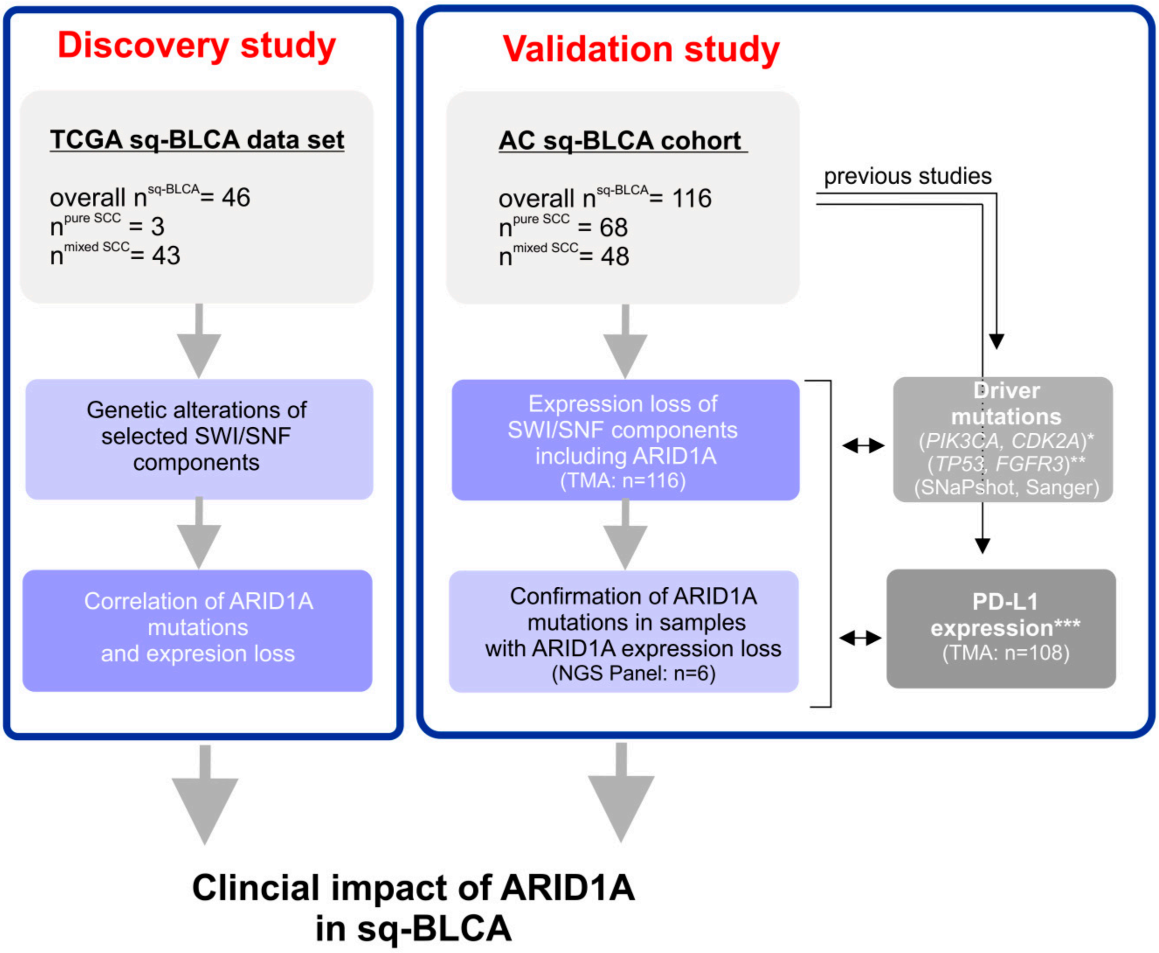

Supplemental Figure 1: Study design of the project. *Left box:* Discovery study. TCGA data sets of carcinomas with histologically squamous differentiation (n=3 pure SCC and n=43 MIX) were analyzed for genetic alterations of seven frequently affected subunits of the SWI/SNF complexes BAF and PBAF. In a second step *ARID1A* mutations were correlated with ARID1A protein expression. *Right box*: Validation study. Protein expression of seven SWI/SNF complex proteins (ARID1A, SMARCA4, SMARCB1, SMARCC1, SMARCC2, SMARCA2 and PBRM1) was assessed by immunohistochemistry in our own sq-BLCA sample cohort. In a second step tumor samples with ARID1A expression loss (IRS0-2) underwent NGS panel sequencing for putative genetic alterations of the *ARID1A* gene. Finally, we analyzed whether a co-occurrence of ARID1A expression loss, genetic alterations and genetic driver mutations (*TP53* and *FGFR3* (*Rose, M. et al. 2020 [5]); *PIK3CA* and *CDKN2A* (**unpublished so far)) and PD-L1 expression (***Morsch, R. et al. 2020 [44]) could be found. In parallel, ARID1A expression loss and genetic alterations were used for both univariate Kaplan-Meier and correlation analysis with clinico-pathological characteristics, however, with no statistical significance.

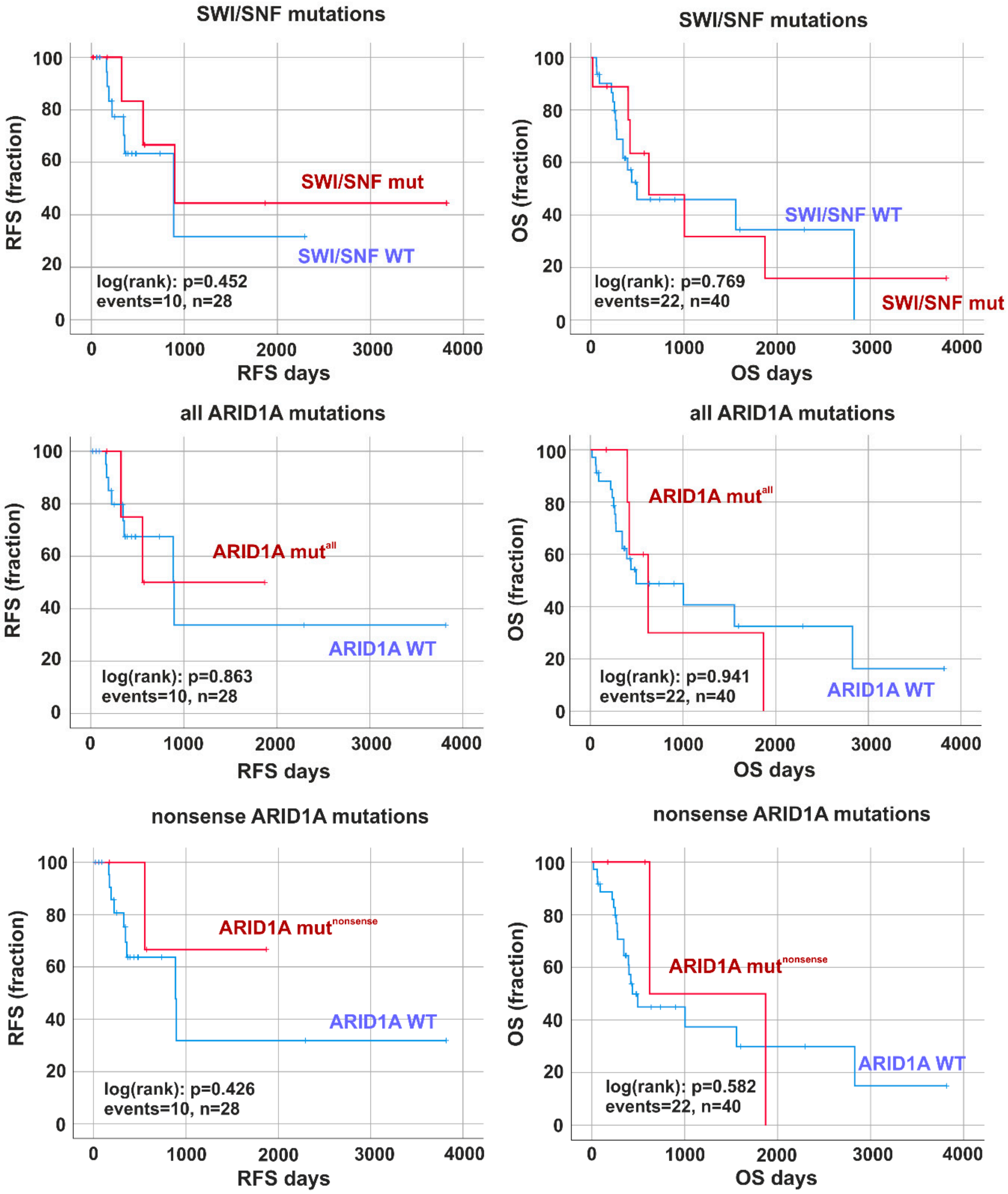

Supplemental Figure 2: Prognostic impact of SWI/SNF and ARID1A mutations on tumor patients' survival. Univariate survival analysis illustrates that tumors with at least a single mutation in one/or more analyzed SWI/SNF components (top row, red curve) or ARID1A mutations (all (middle row) or nonsense (bottom row), red curves) predict no significant shorter RFS / OS compared to the WT status (blue curves, respectively).

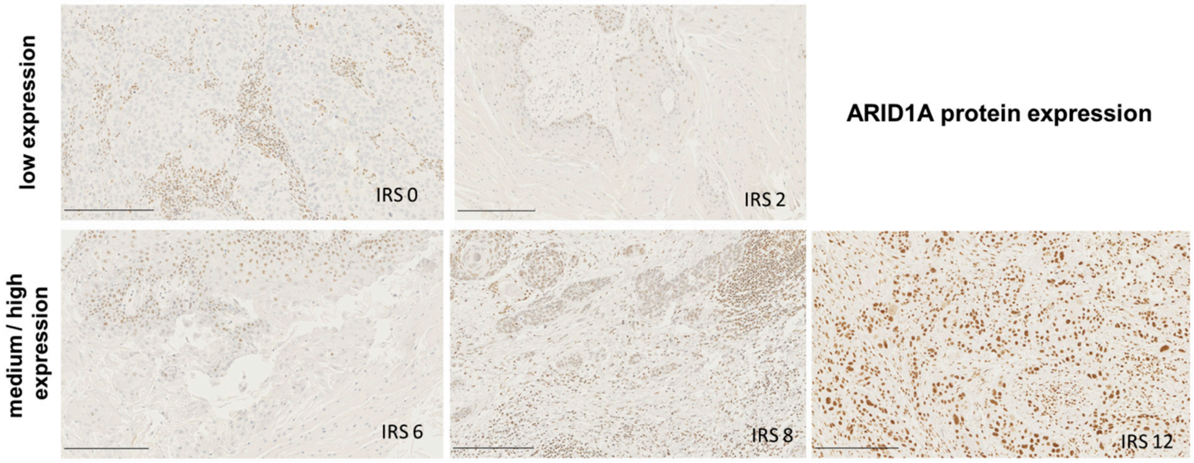

Supplemental Figure 3: Immunohistochemical staining of ARID1A according to calculated IRS. ARID1A staining is shown for IRS 0, 2, 6, 8 and 12 (Black scale bar: 250 μM).

Supplementary Table 1: Primer sequences for Sanger sequencing of FFPE Material.

Gene		Primer sequence	Annealing Temperature
TP53			
	Exon 5	5'-TGCCGT CTTCCAGTTGCTTTATC-3'<br>5'-GCAATCAGTGAGGAATCAGAGGC-3'	60°C
	Exon 6	5'-AGCAGCTGGGGCTGGAGAG-3'<br>5'-CTGGAGGCCCACTGACAAC-3'	63°C
	Exon 7	5'-CCAAGGCGCACTGGCCTCA-3'<br>5'-AGAGGCAAGCAGAGGCTGG-3'	63°C
	Exon 8	5'-CTGATTTCCTTACTGCCTC-3'<br>5'-CTGCACCCTTGGTCTCCTC-3'	60°C
	Exon 9	5'-GTTATGCCTCAGATTCACTT-3'<br>5'-CGGCATTTTGAGTGTTAGAC-3'	55°C
CDKN2A			
	Exon 1	5'-GCTTCCTTTCCGTCATGC-3'<br>5'-CAGGTACCGTGCGACATC-3'	59°C
	Exon 2	5'-CTGTTCTCTCTGGCAGGTCA-3'<br>5'-TGTGCTGGAAAATGAATCCT-3'	59°C
	Exon 2	5'-CTTCCTGGACACGCTGGT-3'<br>5'-TGGAGGCTCTCAGGGTACAAA-3'	59°C

Supplementary Table 2: Clinico-pathological parameters of the TCGA sq-BLCA data set in relation to SWI/SNF mutations.

		SWI/SNF mutations[b]		
	n[a]	**negative**	**positive**	**P-value**[c]
Parameter:				
Tumor stage				
pT2	13	10	3	0.876
pT3-pT4	24	19	5	
Lymph node status				
neg	26	19	7	0.281
pos	10	9	1	

[a]Only patients of the TCGA platform with squamous histology were included; [b]criteria: SWI/SNF mutation is defined if at least one genetic alteration (missense/nonsense) in one/or more of the analyzed components is present; [c]Fisher's exact test

Supplementary Table 3: Clinico-pathological parameters of the TCGA sq-BLCA data set in relation to all identified *ARID1A* mutations.

		ARID1A mutations[b]		
	n[a]	**negative**	**positive**	**P-value**[c]
Parameter:				
Tumor stage				
pT2	13	11	2	0.809
pT3-pT4	24	21	3	
Lymph node status				
neg	26	22	4	0.680
pos	10	9	1	

[a]Only patients of the TCGA platform with squamous histology were included; [b]criteria: genetic alterations of *ARID1A* independent of mutation type; [c]Fisher's exact test

Supplementary Table 4: Clinico-pathological parameters of the TCGA sq-BLCA data set in relation to nonsense *ARID1A* mutations.

		ARID1A mutations[b]		
	***n*[a]**	**negative**	**positive**	**P-value[c]**
Parameter:				
Tumor stage				
pT2	13	11	2	0.239
pT3-pT4	24	23	1	
Lymph node status				
neg	26	24	2	0.825
pos	10	9	1	

[a]Only patients of the TCGA platform with squamous histology were included; [b]criteria: Only nonsense *ARID1A* mutation were included; [c]Fisher's exact test

Supplementary Table 5: Overlap of ARID1A mutations / expression loss with PD-L1 expression.

	**ARID1A mut/del**	**ARID1A exp (IRS≤2)**
**PD-L1 scores for antibody clone 28-8**	**n**	**n**
**IC ≥ 5%[a]**	1/3	2/6
**CPS ≥ 10[b]**	0/3	1/6

[a]atezolizumab , [b]pembrolizumab first line therapy requirement

Supplementary Table 6: Abbreviation list

AKT	serine/threonine protein kinase B
ARID1A	AT-rich interactive domain-containing protein 1A
BAF	BRG1/BRM associated factor
BRG1	alias symbol for SMARCA4
BRM	alias symbol for SMARCA2
cBAF	canonical BRG1/BRM associated factor
CDKN2A	cyclin dependent kinase inhibitor 2A
CNV	copy number variation
COSMIC	Catalogue Of Somatic Mutations In Cancer
CPS	Combined Positivity Score
CXCL13	C-X-C motif chemokine ligand 13
dbSNP	Single Nucleotide Polymorphism Database
DNA	Deoxyribonucleic acid
DSB	double strand break
EMA	European Medicines Agency
F.A.	Fabian Achenbach
FFPE	formalin-fixed paraffin-embedded
FGFR3	fibroblast growth factor receptor 3
HDAC6	histone deacetylase 6
H&E	hematoxylin and eosin stain
HGNC	HUGO Gene Nomenclature Committee (HUGO: Human Genome Organisation)
ICI	immune checkpoint inhibitor
IC(-score)	immune cell score
ID	identification
IRS	Immune Reactive Score
MUT	mutated
ncBAF	non-canonical BRG1/BRM associated factor
NGS	next generation sequencing
NOS	not otherwise specified
N.T.G.	Nadine Therese Gaisa
PBAF	polybromo-associated BRG1/BRM associated factor
PBRM1	Protein polybromo-1
PCR	polymerase chain reaction
PD-L1	Programmed death-ligand 1
PIK3CA	phosphatidylinositol-4,5-bisphosphate 3-kinase catalytic subunit alpha
RWTH	Rheinisch-Westfälische Technische Hochschule
SCC	squamous cell carcinoma
SMARCA2	SWI/SNF related, matrix associated, actin dependent regulator of chromatin, subfamily a, member 2
SMARCA4	SWI/SNF related, matrix associated, actin dependent regulator of chromatin, subfamily a, member 4
SMARCB1	SWI/SNF related, matrix associated, actin dependent regulator of chromatin, subfamily b, member 1
SMARCC1	SWI/SNF related, matrix associated, actin dependent regulator of chromatin, subfamily c, member 1
SMARCC2	SWI/SNF related, matrix associated, actin dependent regulator of chromatin, subfamily c, member 2
SPSS	Statistical Package for the Social Sciences
sq-BLCA	squamous (differentiated) bladder cancer
SWI/SNF	SWItch/Sucrose Non-Fermentable
TCGA	The Cancer Genome Atlas
TMA	Tissue microarrays
TP53	tumor protein p53
UTR	untranslated region
WHO	World Health Organization
WT	wildtype
